# Transforming PKA into PKG – a structure-function approach to understand cyclic nucleotide selectivity

**DOI:** 10.1186/2050-6511-14-S1-P41

**Published:** 2013-08-29

**Authors:** Robin Lorenz, Eui-Whan Moon, Gilbert Y Huang, Albert S Reger, Jeong J Kim, Eugen Franz, Daniela Bertinetti, Choel Kim, Friedrich W Herberg

**Affiliations:** 1 Department of Biochemistry, University of Kassel, Kassel, Germany; 2 Department of Pharmacology, Baylor College of Medicine, Houston, USA; 3 Verna and Marrs McLean Department of Biochemistry and Molecular Biology, Baylor College of Medicine, Houston, USA

## Background

cAMP-dependent protein kinase (PKA) and cGMP-dependent protein kinase (PKG) are the main effectors of distinct cyclic nucleotide pathways and are preferentially activated by cAMP or cGMP, respectively.

We recently characterized the isolated C-terminal cyclic nucleotide binding domain (CNB-B) of the human PKG Iβ as highly cGMP-selective (manuscript in preparation). In a crystal structure of the CNB-B two novel cGMP-specific interaction sites were identified in addition to the previously described threonine residue (T317) in the phosphate binding cassette [1]. Mutation of each individual site resulted in reduced cGMP-selectivity and interfered with cGMP-dependent activation of PKG Iβ.

To gain further insight into the molecular basis of cyclic nucleotide selectivity, we inserted two cGMP-specific interaction sites into the CNB-B of human PKA RIα by mutating corresponding residues. We hypothesize that this way cGMP-specific interaction contacts can be created in PKA and thereby modulate cAMP-selectivity [1,2].

## Results

We characterized a deletion construct of the PKA hRIα CNB-B as cAMP-selective using fluorescence polarization (FP) and surface plasmon resonance (SPR).

In comparison to the wildtype PKA hRIα CNB-B, single mutant constructs showed similar affinities for cAMP- and cGMP-analogs, revealing a loss of selectivity. The combination of two mutations led to a construct with higher affinity for cGMP compared to cAMP.

Co-crystal structures of this double mutant with cAMP or cGMP, respectively, showed that the cGMP-specific interaction contacts retained their function in the context of the PKA hRIα CNB-B.

## Conclusion

The general structure of cyclic nucleotide binding domains is conserved. However, varying amino acids in the binding pocket enable the distinction between cAMP and cGMP. Here we show that cGMP interaction sites found in PKG do restore their specific binding mechanisms when introduced into PKA.

The results underline the relevance of the described novel binding sites in mediating cGMP-selectivity. Still, other features of CNB domains involved in the specific binding mechanism as well as the detailed mechanism of kinase activation need to be investigated.
